# Technical aspects of SEEG limitations and solutions using the Leksell Vantage frame

**DOI:** 10.1007/s00701-025-06662-w

**Published:** 2025-10-27

**Authors:** Insa Prilop, Stephan B. Sobottka, Georg K. Leonhardt, Ilker Y. Eyüpoglu, Witold H. Polanski

**Affiliations:** Department of Neurosurgery, Medizinische Fakultät und Universitätsklinikum Carl Gustav Carus, Technische Universität, Fetscherstraße 74, 01307 Dresden, Germany

## Abstract

**Background:**

Stereo-electroencephalography (SEEG) is an invasive electroencephalography method to precisely locate a seizure onset zone (SOZ). While today´s armamentarium allows any compromise between the historically strict orthogonal and oblique trajectories, the following aspects limit its effectiveness and safety. The shorter the distance from entry to the brain and target the less deviation of the electrode. The closer to orthogonal to the scull the more stable the anchor sits in the scull and deviation can be better controlled. Beyond that, limitations due to the stereotactic frame must be considered. In certain cases, a conflict between frame setup and entry point of the trajectory can arise. This conflict spurred us to explore the limits of stereotactic frame and the associated equipment needed for SEEG.

**Methods:**

We utilize the Elekta Leksell Vantage frame (LVF) and surgical SEEG-instruments of the company AD-Tech. Planning of the trajectories is performed with a software based on MRI scans. After co-registration with stereotactical CT-angiogram coordinates for the centre-of-arc-principle to set up the LVF are generated. The frame setup focuses on the orientation of the ring-shaped z-axis, which impact SEEG electrode placement. Key factors, such as arc-angle and x-coordinate, influence instrument positioning and potential interference with the frame. Various combinations of ring-scale direction, arc alignment, arc-angle, and x-coordinate were attempted, along with the limitations of the ring angle in relation to y- and z-axis.

**Results:**

Since 2018 our department performed 19 SEEG-implantations of in total 188 SEEG-electrodes. The average age at presentation of the 15 male and 4 female patients was 42 years (range 28 to 58). In 6 of the 19 implantation the ZD Inomed frame was used, while the other 13 cases were performed with the LVF. An average of 9 electrodes were applied using the ZD Inomed frame compared to 11 electrodes with the LVF. Both approaches aimed for bihemispheric targets. The average time of implantation of a single electrode using the ZD Inomed frame was 20,7 min, while the LVF took 4 min less (16,6 min). Other factors such as electrode repositioning or deviation and postoperative complications occurred very rarely. In at least 12 cases concerning 19 out of 188 (10.1%) electrodes an intraoperative trajectory replanning became necessary because of conflict between frame setup and entry point of the trajectory.

**Conclusion:**

Our clinical experience confirmed that the complex planning of SEEG trajectories occasionally leads to a conflict between frame setup and entry points. The systematic analysis of the utilized stereotactic frame and SEEG-instruments can prevent replanning during surgery and improves patient’s safety and quality management.

**Supplementary Information:**

The online version contains supplementary material available at 10.1007/s00701-025-06662-w.

## Introduction

Stereo-electroencephalography (SEEG) with implanted depth electrodes supplements non-invasive scalp electroencephalography (EEG) in patients with pharmaco-resistant epilepsy to precisely locate the seizure onset zone (SOZ) and reveal the three-dimensional epileptogenic network. Beyond diagnostic purposes leading to resective surgery it can be employed for radiofrequency thermocoagulation.

Planning intracerebral electrodes using a stereotactic frame is an interdisciplinary endeavour, involving a neurosurgeon and a neurologist. Three-dimensional magnetic resonance imaging (MRI) is mandatory to delineate avascular and cortical trajectories confirming or debunking the neurologist’s working hypothesis, based on seizure semiology and scalp EEG. Typically, 6–15 electrodes are used, with at least a few contacts placed in the suspected SOZ and others surrounding it to outline the resection margin [4, 5]. Frequently used stereotactic frames are the ZD Inomed frame (Inomed GmbH, Emmendingen, Germany) and the Leksell Vantage frame (Elekta, Stockholm, Sweden).

Commonly used SEEG-electrodes are flexible, semi-rigid, round tipped and contain between 5–18 contacts (2mm contact length) with various spacing of 2-5mm and a diameter of 0,86mm. Implantation is performed under general anaesthesia using either frame-based or frameless technique. Overall, it is not possible to generalize a reasonable comparison of the duration of surgery of each technique, because of varying experiences and number of procedural steps performed in different centres. Although frame-based placement accuracy is difficult to compare with frameless techniques in terms of heterogeneity of number of cases, statistics and experience of centres are described in a meta-analysis by Vakharia et al. [2, 8].

Historically, SEEG targets regions predominantly within the temporal lobe. During the last years the indication for SEEG-implantation expanded and target regions became more complex, relaying on results of MRI-negative and PET-positive intracerebral regions, as extratemporal regions, especially the insula [3, 6]. While planning, trajectories should ideally be perpendicular to the skull and cortex surface to minimize electrode deviation and prevent drill bit skiving. Moreover, the risk for electrode deviation increases with longer transcerebral trajectories.

Entry points anterior to the hairline are avoided for cosmetic reasons, with exceptions of targeting the anterior insula, especially in children via frontal entry points using oblique instead of orthogonal trajectories due to a better coverage of the insula and lower risk of vessel injury. Occipital approaches could lead to pressure points when lying supine [5]. To reduce the 1% risk of clinically relevant haemorrhage, which is stated by a systematic review of 57 articles by Mullin et al., trajectories should avoid sulci, which mostly contain vessels, as well as areas with brain atrophy leading to subdural space and previous surgical cavity [7]. Bleedings are probably underreported as some publications of SEEG procedures do not mention any complications. Besides, all vessels along the planned electrode path should be bypassed.

Stereotactic frame placement can occasionally lead to conflicts between frame setup and the entry point of the trajectory, especially for the temporal approach. This conflict spurred us to explore limitations of the stereotactic frame and the associated equipment needed for SEEG. Restrictions by the frame can require adjustments of the trajectory mid-surgery, increasing operating time. Therefore, we conceived a preoperative planning routine for the Leksell Vantage frame system that avoids restricted lead positions, ensuring safe and efficient electrode placement.

## Methods

In our department, we use the stereotactic Leksell Vantage frame (Elekta, Stockholm, Sweden) and surgical SEEG-instruments from AD-Tech Medical Instrument Corporation (AD-Tech Medical Instrument Corporation Oak Creek, United States). This carbon frame, released in 2018 represents a lighter and MRI-compatible upgrade of the previous model (Leksell Stereotactic System). The former is achieved by the use of innovative materials that minimize magnetically-induced radiofrequency heating 8. https://www.elekta.com/products/neurosurgery/leksell-vantage-stereotactic-system/.

The exact target planning is performed by the neurosurgeon and the neurologist using Elements software (BrainLab AG, Munich, Germany) and 3-D T1-weighted and FLAIR- MRI scans (3 Tesla, Siemens, Munich, Germany). After general anaesthesia, the Leksell Vantage frame is mounted, followed by a stereotactic computer tomography (CT) angiogram. Co-registration of the preoperative MRI and CT-angiogram generates the necessary coordinates. In a sterile setting, a small incision is made using a Kirschner wire, followed by a craniotomy with a 3 mm drill bit (Aesculap, Tuttlingen, Germany**)**. This drill bit is stabilized by a holder attached to the Leksell Vantage arc. Dura coagulation is performed with a monopolar coagulating fibre **(**Aesculap, Tuttlingen, Germany). Then, bone anchors (AD-Tech Medical Instrument Corporation Oak Creek, United States) in different sizes are screwed into the skull. The electrode´s insertion angle and bone thickness are measured using CT and MRI scans. A stylet is inserted following the planned trajectory and after removal, the electrode is inserted into the established canal. This procedure is replicated for each electrode. A post-implantation CT-scan verifies the accurate electrode placement and reveals potential complications like bleeding. All surgeries were performed by an established team of two neurosurgeons (SBS and WHP) and a neurologist (GKL).

### Setup of the stereotactic Leksell Vantage frame

The initial setup is based on lubricated components to ensure smooth operation. In a sterile setting, the first step involves fastening the y-axis to the frame holder attached to the frame on the patient’s head, ensuring the correct orientation marked as “anterior”. The z-axis connects to the y-axis with their ring-shaped part oriented either forwards or backwards, influencing the accessibility for electrode placement. The semi-circular arc, containing the x-axis and instrument carrier, positioned in a certain arc-angle, is placed on the ring-shaped part of the z-axis in a certain ring-angle depending on the planned approach (lateral-left or lateral-right).

**Fig. 1:**
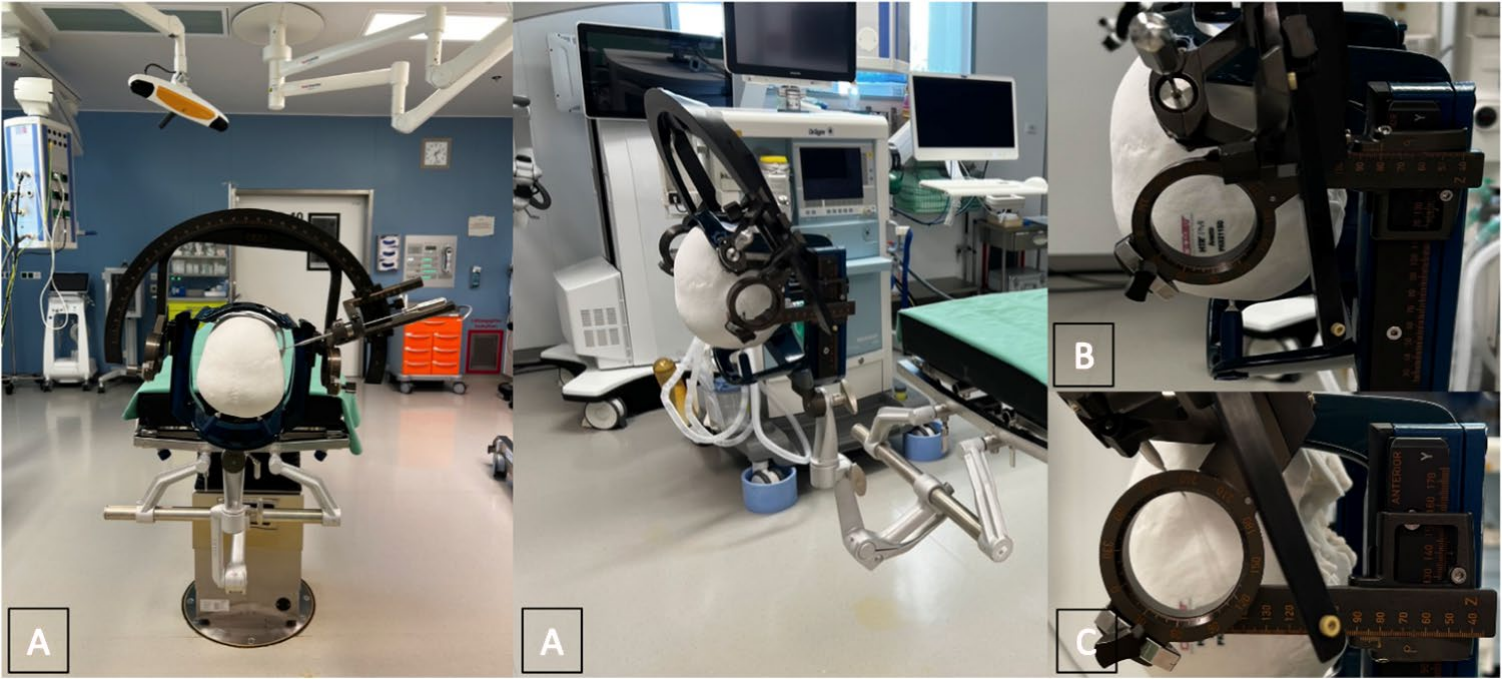
Image **A** shows the setting in the operating theatre with the Leksell Vantage frame with SEEG tools attached to the patient’s head. Image **B** and ***C*** outline the two different options of the setup of the ring-shaped part of the z-axis, facing backwards (*B*) and forward (*C*)

**Fig. 2:**
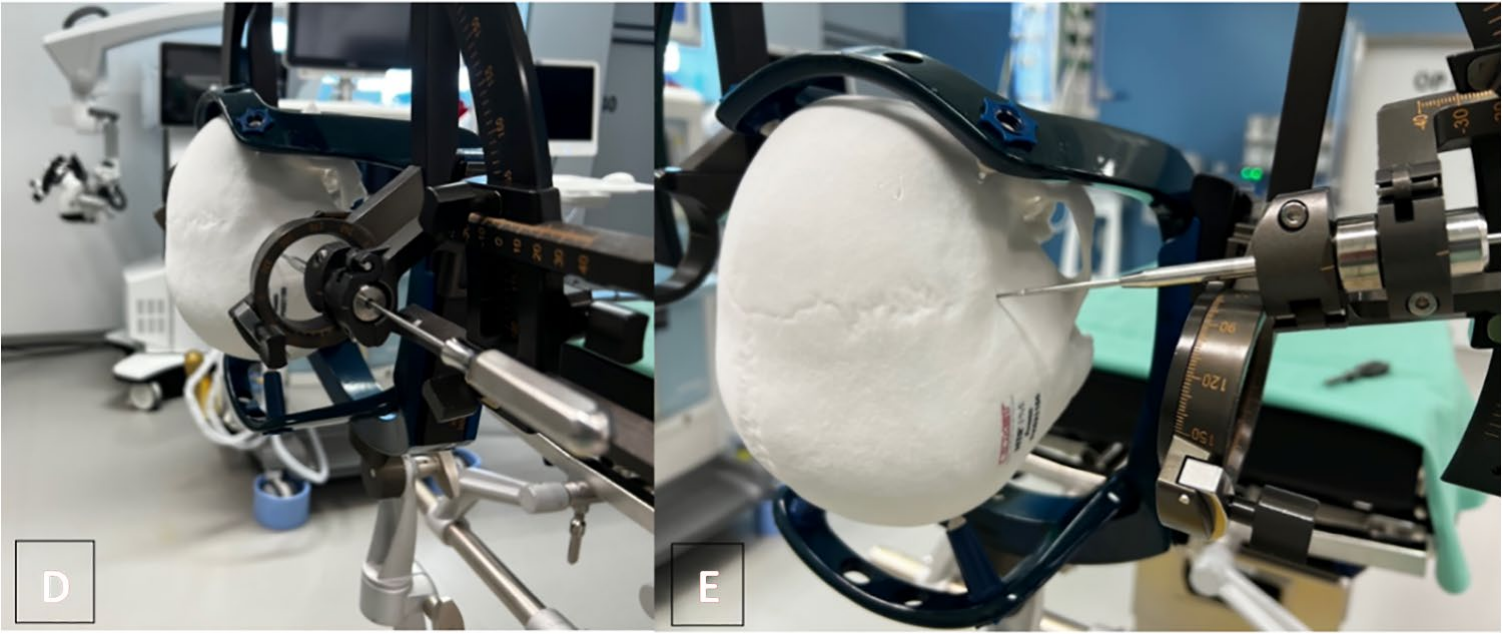
Image **D** depicts the possibility of executing a trajectory through the ring-shaped part of the z-axis. Whereas Image **E** presents a possible conflict of the frame setting and SEEG tools in terms of bordering on collision

The arc-angle and x-coordinate are crucial because they can limit the positioning of the instruments, in a way that improper alignment can interfere with the frame setup. Besides, adjustment in ring-angle in dependence on y- and z-coordinates can affect electrode placement with limitations through the frame. Furthermore, there is the option to plan trajectories through the ring-shaped part of the z-axis for temporal approaches. Various combinations of directions of the ring-shaped z-axis, alignment of the semi-circular arc, arc angle and x-coordinate are tested to determine an optimal setting for the electrode insertion. The intraoperative setup is shown in Figs. 1 and 2*.*

The Leksell Vantage frame system limits depending on the y- and z-coordinates within the 360° of the ring-angle range, which varies depending on the x-coordinate. Therefore, we also analysed these parameters to maximize the potential for precise electrode placement and minimize interference.

### Examination of accuracy of electrodes

To compare the accuracy of both frame systems, we used the following equation to analyse the deviation of the implanted electrode from the planned trajectory in relation to the coordinates of the electrode and the trajectory tip: $\sqrt{\left\{{\left({x}_{2}- {x}_{1}\right)}^{2}+ {\left({y}_{2}- {y}_{1}\right)}^{2}+ {\left({z}_{2}- {z}_{1}\right)}^{2}\right\}}$. For statistical analysis, we used a nonparametric Mann–Whitney-U-test of the null hypothesis and a nonparametric Kolmogorov–Smirnov-test for one-dimensional probability distribution.

### Patient cohort

19 patients with a total number of 188 SEEG-electrodes were implanted. Clinical and technical data are presented in Table 1.

**Table 1:**
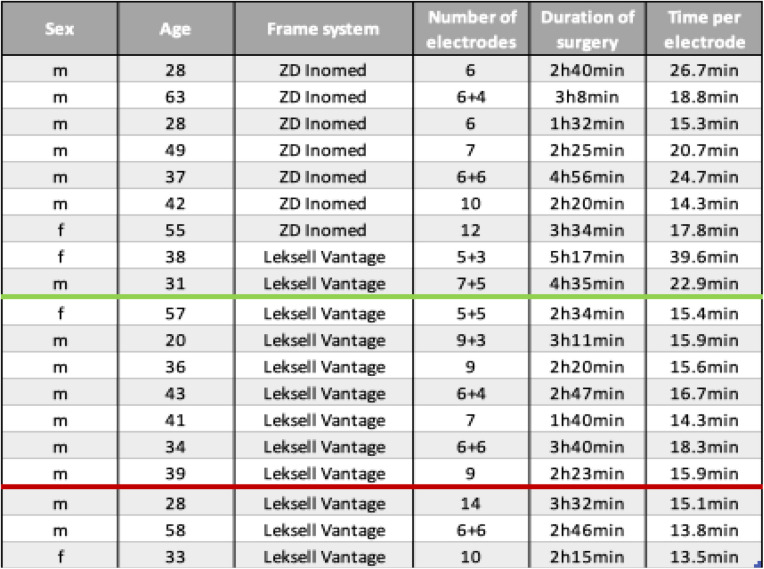
Overview of the cohort with SEEG implementation

Sex: m=male and f=female. The age is stated in years at the date of surgery. The number of electrodes defines the sum of the electrodes of the left hemisphere + the electrodes of the right hemisphere. The duration of surgery describes the period of the surgeons work in the operating theater from first cut to the last stitch. The time per electrode is given in minutes (min). The first 9 of the table underwent surgery without use of any of the developed illustrations and tables to avoid restrictions. The following 7 patients underwent surgery using the scheme affecting the arc angle and the x-coordinate. For the remaining 3 patients, both schemes were established during surgery

## Results

19 patients with a total number of 188 SEEG-electrodes were implanted. Clinical and technical data are presented in Table 1. The first 9 patients underwent surgery without the use of the proposed guidelines to avoid certain restrictions. The following 7 patients underwent surgery using the scheme affecting the arc angle and the x-coordinate. For the remaining 3 patients both schemes were applied during surgery. The average age at surgery of the 15 male and 4 female patients was 42 years. In 7 out of these 19 cases, the ZD Inomed frame (Inomed GmbH, Emmendingen, Germany) was used, while the remaining 12 cases were performed with the Leksell Vantage frame. An average of 9 electrodes were applied using the ZD Inomed frame compared to 11 electrodes with the Leksell Vantage frame. In total, 188 electrodes were implanted, 63 electrodes using the ZD Inomed frame and 125 using the Leksell Vantage frame. The average implantation time per electrode was 20.7min using the ZD Inomed frame, versus 16.6min with the Leksell Vantage frame (details in Table 2). Implanted electrodes showed an average deviation at the target of 2.6mm with a median of 2.0mm and a range of 0 to 9.3mm using the ZD Inomed frame and a significant lower average deviation of 1.7mm planned trajectory with a median of 1.3mm and a range of 0 to 13.1mm (*p* = 0.009) for the Leksell Vantage frame. The data of deviations were not normally distributed (*p* < 0.001).

**Table 2:**
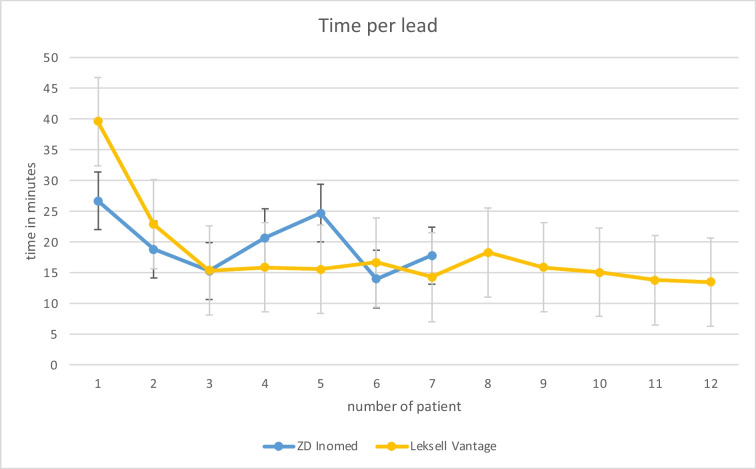
Comparison of the mean surgery time needed for each electrode in comparison of both stereotactic frame systems

There were two intra- and postoperative complications. In one case a postoperative bleeding along the puncture trajectory led to mild hemiparesis and aphasia, both had improved before discharge. A second patient had an acute kidney failure due to paracetamol intolerance. Her problem resolved swiftly and completely. Notably, in 12 cases involving at least 19 of the 188 (10.1%) electrodes, intraoperative trajectory replanning was necessary due to conflicts between the frame setup and entry point of the trajectory, which extended surgery- and anaesthesia time.

Additionally, the development of time per lead in both frame systems is illustrated in Table 2*.*

The required intraoperative adjustments to the trajectories, due to conflicts with the frame, primarily were related to the arc angle and the x-coordinate. To avoid intraoperative replanning, overview tables (Table 3, Electronic Supplementary Materials Tables 5–7) were created to identify potential conflicts between the frame and trajectories during preoperative planning. This table illustrates two alignments of the semi-circular arc (lateral left and – right). For each, it highlights unfeasible positions when the ring-shaped z-axis face forwards or backwards. Table 3 is an excerpt of the overall schedule, which is added as Electronic Supplementary Material (Tables 5*–*7).

**Table 3:**
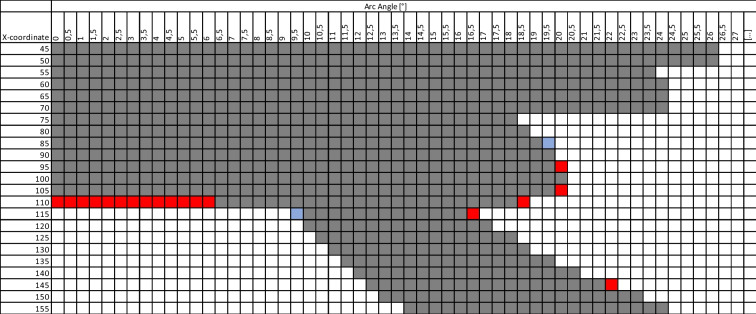
Lateral left – arc setting. Depiction of arc angle on the x-axis and x-coordinates of the frame on the y-axis. Grey-shaded windows represent a conflicting setting of the electrode-trajectory and frame setting for both montage directions of the ring-shaped part of z-axis (facing forward or backward). **Red**-shaded windows represent settings which will not conflict when the ring-shaped part of z-axis facing **forward**, but are not working for the backwards setting. The **blue**-shaded windows represent settings which will not conflict when the ring-shaped part of z-axis facing **backwards**

**Table 4:** Illustration of the range of arc angle depended on y- and z-coordinates for feasible adjustment options. X-coordinate is at a maximum of 155 mm, arc angle is 0°

y-axis (mm)	z-axis (mm)	Ring-angle (°)—lateral left	Ring-angle (°)—lateral right
25–75	35–70	307–153	339–195
25–75	71–120	293–137	327–3
25–75	121–159	127	-
76–125	35–70	315–193	313–191
76–125	71–120	319–179	317–179
76–125	121–159	319–177	135–269
126–175	35–70	343–211	283–161
126–175	71–120	341–120	283–147
126–175	121–159	359–211	267–133

**Fig. 3:**
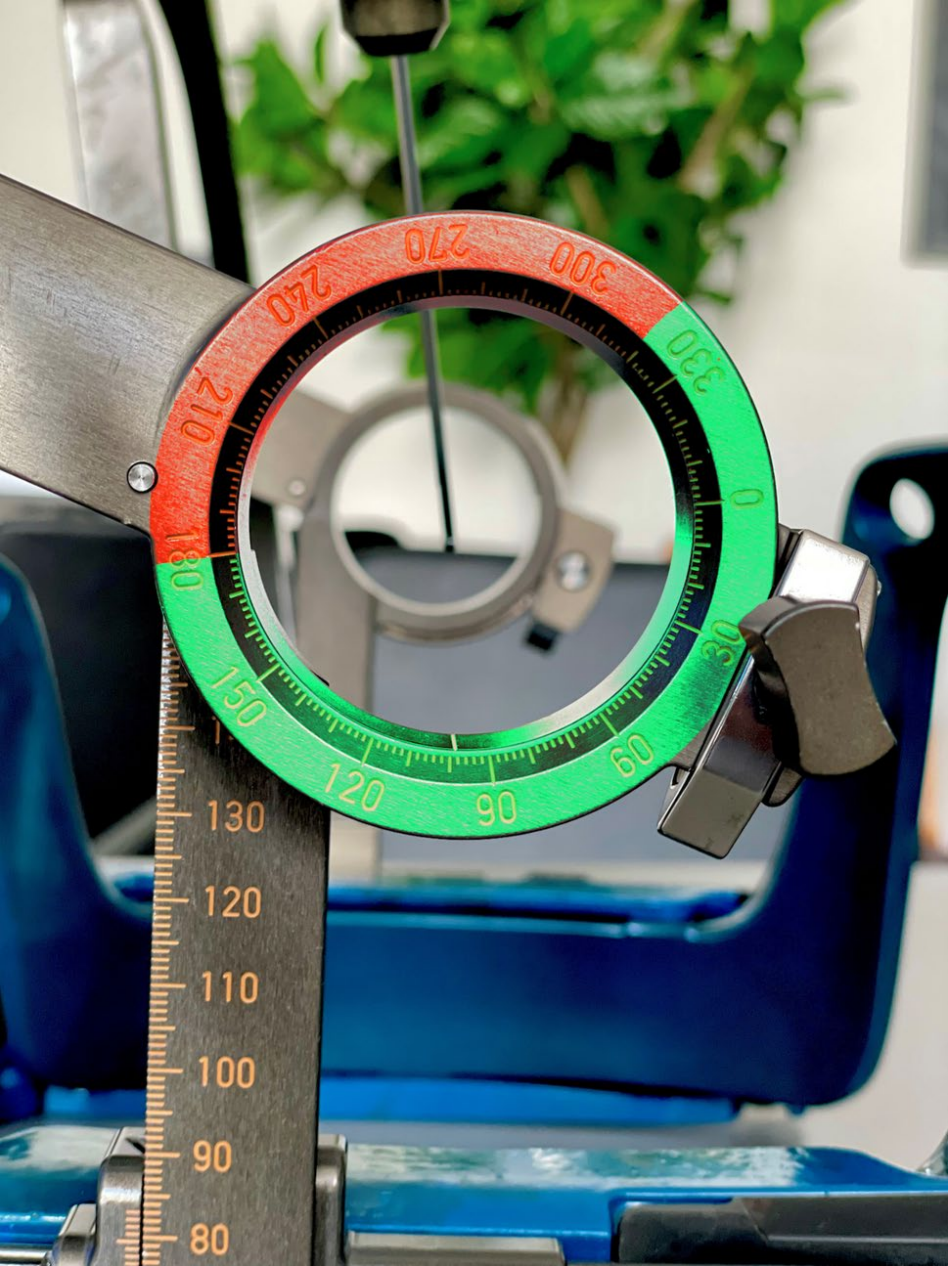
Exemplary representation of technically feasible (green) and not feasible (red) adjustment options for the ring angle at the most commonly used y-coordinates (76-125mm) and z-coordinates (71-120mm). Note that only stereotactic system-related limitations and not patient-dependant constraints were considered

The examination of the limitations associated with the ring angle revealed that, in addition to the y- and z-coordinates, the x-coordinate also bears potential conflicts. For further investigation and clarity, x-values were grouped into categories: those between 76 to 122 mm were classified as the low limitation group, while the remaining values were classified as the minimum/maximum group with large limitations. Presuming that most approaches aim for a temporal entry point, the arc angle was set to 0°. The following table (Table 4) presents the range of the ring angle dependent on the y- and z-coordinates to ensure no conflicts between stereotactic frame and planned trajectory. The Fig. 3 shows the technically feasible and not feasible adjustment options for the ring angle at the most commonly used y-coordinates (76–125 mm) and z-coordinates (71-120mm). To ensure a clearer overview, coordinates were combined into sections. The additional schedule is provided in the supplement (*Table* *8* and *Table* *9*). After these overviews were applied, no intraoperative replanning of trajectories was necessary in any case.

**Fig. 4:**
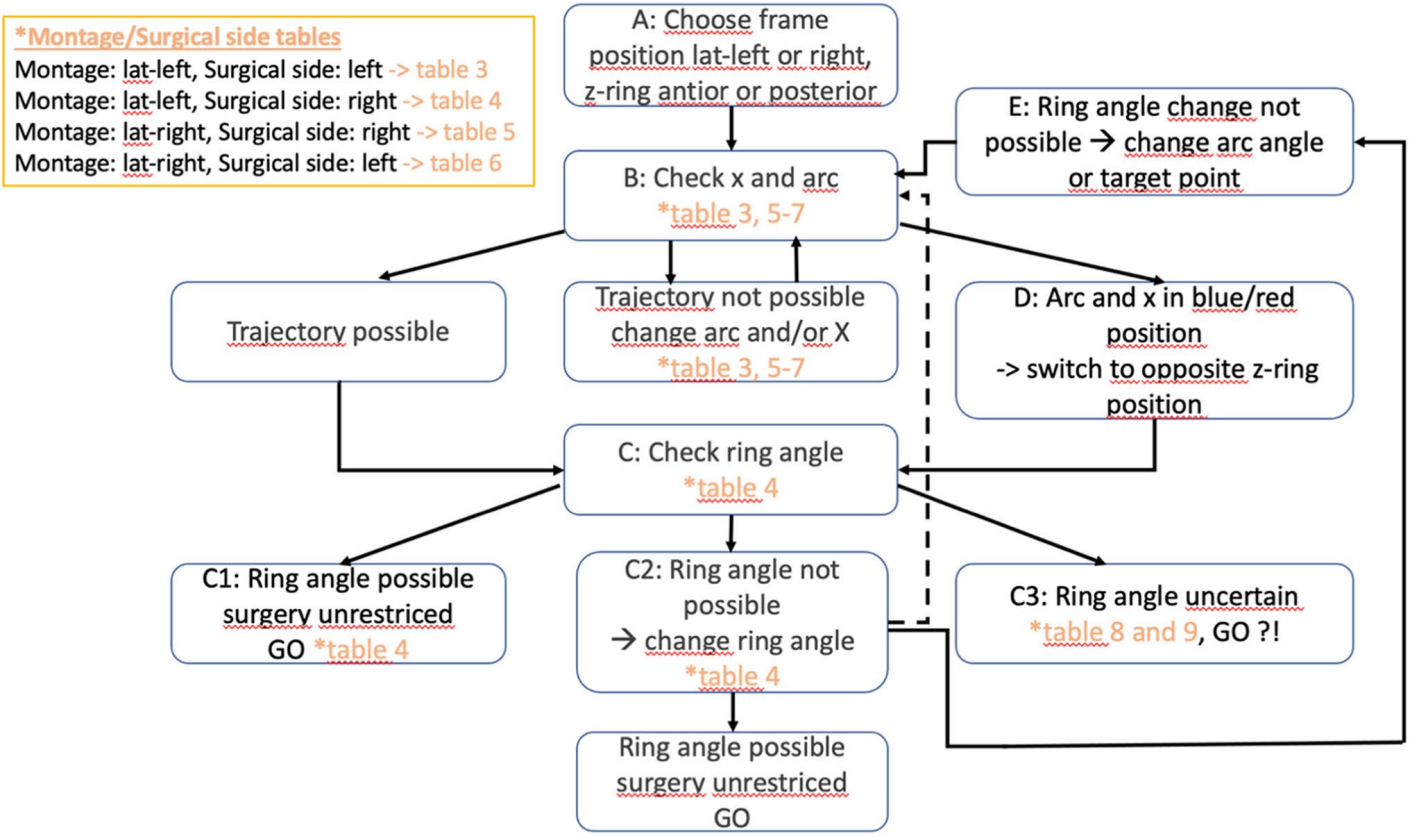
Review and verification of the target and trajectory frame values for an electrode: A) Choose a possible stereotactic frame position (lateral left or right, Z-ring ant. or post. B) Check x-coordinate and arc-angle of trajectory using the appropriate table (Table 3, Supplementary Material Tables 5–7) depending on type of frame montage and surgical side*. C) If trajectory is possible, check for feasible ring angle based on y- and z-coordinates using Table 4 (continue C1/C2). C1) If ring angle is possible (Table 4), surgery will be unrestricted (GO). C2) If ring angle is not possible (Table 4), re-planning of trajectory is recommended preferentially by changing ring angle to appropriate range using Table 4 (GO) C3) Otherwise the frame values may be checked with *Table* *8* and *9* for definitely impossible trajectories (Supplementary Material Table 8) or potentially possible trajectories (Supplementary Material Table 9). D) If trajectory is in a blue or red position, you may switch to the opposite z-ring position (continue C). E) If trajectory is NOT possible, change X and/or arc to the next possible location using the appropriate table from (Table 3, Supplementary Material Tables 5–7); arc angle might be preferable to preserve the target point (continue C)

To ensure a smooth and standardized procedure of trajectory planning we developed an assisting workflow (Fig. 4).

## Discussion

### Summary of key findings and comparison with existing literature

Using different frame systems to apply SEEG-electrodes affords a systematic schedule to represent conflicts between frame setting and electrode trajectory. A commonly used frame is the ZD Inomed frame. Our experiences showed that using the ZD Inomed frame has the advantage of lower attachment on the patient’s head, facilitating easier access to the temporal lobe. However, a disadvantage of this single-arm frame is greater instability during stereotactic drilling, which resulted in statistically significant difference in the accuracy of electrodes implanted using the ZD Inomed frame. By utilizing the attached tables to verify the planning of trajectory coordinates at the entry point using the Leksell Vantage frame before starting the surgery, the average time needed per electrode was reduced to 16.6 min. This is more efficient compared to the average time of 26.5 min per electrode reported in comparative literature as the meta-analysis by Vakharia et al. [8]. The comparison of actual electrode accuracy at the target point showed a median of 1.7 mm deviation using the Leksell Vantage frame and a median of 2.0 mm using the ZD Inomed frame. There is a lack of direct comparison in existing literature regarding target accuracy of these two frame systems when used for SEEG implantation. Van der Loo et al. described the in vivo accuracy of the Leksell Vantage frame, noting a median target error of 2.69 mm for 517 SEEG-electrodes [9]. When comparing the Leksell Vantage frame with its predecessor, the Leksell G-frame, an important consideration is that the Leksell G frame allows the aiming arc to pivot about transverse or sagittal axis. Both frames possess a comparable degree of submillimetre stability, making them suitable for high-precision stereotactic procedures [1]. However, the Leksell Vantage frame enables the most artifact-free acquisition of stereotactic MR images.

Furthermore, SEEG implantation can be performed using robotic systems, which offer greater flexibility with respect to mechanical and physical constraints when trajectory planning flexibility is limited, although the available on their accuracy remains limited [8].

### Strength and limitations

A major strength of this technical report is its pioneering description of the technical limitation of the Leksell Vantage frame when used for SEEG, especially for temporal trajectories. This data provide a valuable practical guide to navigate the electrode planning workflow, helping to prevent intraoperative conflicts with the stereotactic frame setting and ultimately improving patient safety by reducing surgery and anaesthesia duration.

However, several limitations need to be mentioned. The small sample size limits generalizability of the clinical findings and weakens the statistical power. Additionally, as patients were not randomized to either frame system, confounders such as choice of the surgeons due his or her preference or experience could not be excluded. The sequential nature of cases also cannot account for potential confounding effect of surgeons learning curve. Furthermore, our experience in this cohort is limited to two stereotactical systems without comparison of additional established stereotactic device, such as Inomed Riechert-Mundinger), the Integra Radionics Brown-Robert-Wells and Cosman-Robert-Wells, and the widely used Leksell G-frame.

### Clinical implications

Our clinical experience has shown that the complex planning of SEEG trajectories can sometime result in conflicts between the frame setup and their entry points. Systematic exploration of the stereotactic frame and SEEG-instruments, particularly the use of the Leksell Vantage frame, not only helps in preventing mid-surgery replanning but also significantly reduces operation time, enhancing overall efficiency and patient safety.

The surgeons found it very helpful to use the created tables during the planning phase to adjust trajectories and the corresponding targeted structures in accordance with the operation.

### Future directions

Building upon our preliminary findings, future research should aim to validate the utility and efficiency of the developed planning workflow and conflict-avoidance tables in larger, prospective patient cohorts across multiple centres. A multicentre, randomized study would be particularly valuable to assess generalizability and to control for confounding factors such as surgeon experience and patient anatomy. Furthermore, the integration of artificial intelligence (AI)-based planning tools may enhance preoperative trajectory simulations by predicting frame conflicts and optimizing electrode configurations in real-time. Combining this with patient-specific 3D modeling could allow for more precise visualization of individual anatomical constraints and help tailor surgical strategies. Finally, further development and standardization of training protocols incorporating this workflow might reduce the learning curve and promote wider adoption of the Leksell Vantage system in SEEG procedures, ultimately improving patient safety and procedural outcomes.

## Supplementary Information

Below is the link to the electronic supplementary material.

Supplementary file1 (PDF 943 KB)

## Data Availability

No datasets were generated or analysed during the current study.
