# Supplementary material for: Technical aspects of SEEG limitations and solutions using the Leksell Vantage frame
Source: Acta Neurochir (Wien). 2025 Oct 27;167(1):282. doi: 10.1007/s00701-025-06662-w (PMC12559164; doi:10.1007/s00701-025-06662-w)
Supplement: Supplementary file 1 — Supplementary file1 (PDF 943 KB) [file 701_2025_6662_MOESM1_ESM.pdf]

Table 5

151	151,5	152	152,5	153	153,5	154	154,5	155	155,5	156	156,5	157	157,5	158	158,5	159	159,5	160	160,5	161	161,5	162	162,5	163

Table 6

X-coordinate [mm]	0	0,5	1	1,5	2	2,5	3	3,5	4	4,5	5	5,5	6	6,5	7	7,5	8	8,5	9	9,5	10
45																					
50																					
55																					
60																					
65																					
70																					
75																					
80																					
85																					
90																					
95																					

100																					
105																					
110																					
115																					
120																					
125																					
130																					
135																					
140																					
145																					
150																					
155																					

Table 7

150	151	151	152	152	153	153	154	154	155	155	156	156	157	157	158	158	159	159	160	160	161	161	162	162

Table 8

y-axis (mm)	z-axis (mm)	ring-angle (°) - lateral left	ring-angle (°) - lateral right
25-75	35-70	154-306	196-338
25-75	71-120	138-292	4-348
25-75	121-159	126-16	212-18
76-125	35-70	194-314	192-312
76-125	71-120	180-318	180-316
76-125	121-159	178-318	178-314
126-175	35-70	212-342	162-282
126-175	71-120	148-2	148-4
126-175	121-159	212-8	134-266

Table 9

y-axis (mm)	z-axis (mm)	ring-angle (°) - lateral left	ring-angle (°)
25-75	35-70	-	-
25-75	71-120	-	326-347
25-75	121-159	127-14	213-17
76-125	35-70	-	-
76-125	71-120	-	-
76-125	121-159	-	315-134
126-175	35-70	-	-
126-175	71-120	121-147	5-282
126-175	121-159	0-7	-
